# Incidental Detection of a Chromosomal Aberration by Array-CGH in an Early Prenatal Diagnosis for Monogenic Disease on Coelomic Fluid

**DOI:** 10.3390/life13010020

**Published:** 2022-12-21

**Authors:** Margherita Vinciguerra, Filippo Leto, Filippo Cassarà, Viviana Tartaglia, Michela Malacarne, Domenico Coviello, Valentina Cigna, Emanuela Orlandi, Francesco Picciotto, Gaspare Cucinella, Emanuela Salzano, Maria Piccione, Aurelio Maggio, Antonino Giambona

**Affiliations:** 1 Unit of Molecular Diagnosis of Rare Hematological Diseases, Azienda Ospedaliera Ospedali Riuniti Villa Sofia-Cervello, 90146 Palermo, Italy; 2 Laboratory of Human Genetics, Istituto Giannina Gaslini, 16147 Genoa, Italy; 3 Unit of Fetal Medicine and Prenatal Diagnosis, Azienda Ospedaliera Ospedali Riuniti Villa Sofia-Cervello, 90146 Palermo, Italy; 4 Medical Genetics Unit, Azienda Ospedaliera Ospedali Riuniti Villa Sofia Cervello, 90146 Palermo, Italy; 5 Unit of Hematology for Rare Diseases of Blood and Blood-Forming Organs, Azienda Ospedaliera Ospedali Riuniti Villa Sofia-Cervello, 90146 Palermo, Italy

**Keywords:** prenatal diagnosis, array comparative genomic hybridization, coelocentesis, monosomy X, beta thalassemia

## Abstract

Background: Turner syndrome is a rare genetic condition in which a female is partly or completely missing an X chromosome. Signs and symptoms vary among those affected. In fetuses that survive at birth and without congenital malformations, the prognosis is usually positive, but it has high lethality in utero, especially in the first trimester of pregnancy. Methods: We report a case of monosomy X detected during a prenatal diagnosis for beta thalassemia on coelomic fluid (CF) at the VIII week of gestation. Beta globin gene analysis, whole genome amplification (WGA), quantitative fluorescent PCR and array comparative genomic hybridization (array-CGH) were performed on DNA extracted from CF. Results: A monoallelic pattern of all Short Tandem Repeats mapped on the X chromosome was found and array-CGH performed on WGA from a few fetal erythroblasts confirmed monosomy X. Conclusion: This report underlines the importance of an early prenatal diagnosis and the countless potentialities of array-CGH that could make definition of molecular karyotype possible from a few fetal cells, unlike conventional cytogenetic techniques that require a greater cellular content. This is the first report of a molecular karyotype obtained from two cells selected by micromanipulation of CF and defined at such an early gestational age.

## 1. Introduction

Turner syndrome is a rare condition that occurs in one out of every 2500 to 3000 live female births and is characterized by the partial or complete absence of one X chromosome (45,X karyotype), often in mosaic karyotypes. Signs and symptoms vary among those affected. Morbidity and mortality are increased in women with Turner syndrome compared with the general population and the involvement of multiple organs through all stages of life necessitates a multidisciplinary approach to care. Despite an often-evident phenotype, the diagnostic delay can be substantial and the average age at diagnosis is around 15 years of age.

A diagnosis of Turner syndrome can be confirmed with standard karyotyping. More than one half of patients with the condition will have a missing X chromosome (45,X) in all cells studied or a combination of monosomy X and normal cells (45,X/46,XX; mosaic Turner syndrome). A mosaic result does not necessarily predict severity because karyotyping only investigates lymphocytes, not the relevant tissues (e.g., brain, heart, ovaries) [1].

In prenatal diagnosis, some ultrasound abnormalities may lead to suspicion of a monosomy X: hygroma (head and neck), fetal hydrops, coercedation of the aorta, hydronephrosis, short femur and homer. A karyotype on chorionic villi or amniotic fluid may be performed or FISH in fetuses who died in utero [2]. In fetuses that survive at birth and in the absence of congenital malformations, the prognosis is usually positive, but it has high lethality in utero (approximately 75%) especially in the first trimester of pregnancy, so the exact incidence is underestimated because these cases will never be diagnosed, unless there is a suspicion, and a cytogenetic analysis is carried out on the abortive material.

Couples at risk of generating a fetus suffering from monogenic disease have the opportunity to undergo villocentesis (XI-XIII week of gestation) or amniocentesis (XVI week of gestation) in order to know the genetic structure of the fetus. In our department, it is possible to perform sampling and analysis of coelomic fluid (CF) between VII and IX week of gestation. Coelocentesis is a reliable and safe procedure [3,4] and, thanks to its precocity, aneuploidies on chromosomes 13, 18, 21, X and Y can be identified earlier than standard procedures such as villocentesis and amniocentesis [5]. Until now, no published report of a possibility to obtain a conventional karyotype is available due to the exiguity of fetal cells in CF.

The extra-celomic cavity develops at IV week of pregnancy within the extra-embryonic mesoderm. It surrounds both the fetus and amniotic cavity reaching maximum volume at VII–IX weeks, then it disappears at XII–XIII weeks. Inside the celomic cavity, the secondary yolk sac is present as an independent floating organ. The mesoderm layer of the yolk sac produces the first blood cells within the blood islands and successively hematopoietic progenitor cells complete their maturation in the bloodstream. Coelomic fluid can be considered as an ultra-filtrate of maternal serum, containing a high protein concentration, urea and many other molecules. It is an important transfer interface and a reservoir of nutrients for the embryo. Coelomic fluid contains fetal cells that can be used for prenatal diagnosis of monogenic diseases in an earlier gestational period than villocentesis and amniocentesis. Using high-resolution ultrasound transducers, it is possible to clearly identify the coelomic cavity and by transvaginal or transabdominal insertion of a needle into the extra-amniotic cavity, it is possible to aspirate CF at VII–IX weeks of gestation. Previous studies [6,7,8,9,10,11] reported difficulties using coelomic fluid for a prenatal diagnosis because of the high grade of maternal contamination in fetal DNA interfering with the interpretation of molecular data and the relatively low number of fetal cells in CF. Coelomic fluid presents a great variability of cell density and types characterized by different morphology. A variable percentage of maternal and fetal cells from one sample to another was observed reflecting the different percentage of maternal contamination. The use of micromanipulator has fixed the issue of maternal contamination in a pauci-cellular fluid as CF [12].

We report a case of monosomy X detected during a prenatal diagnosis for beta thalassemia and the definition of a new experimental application to overcome the impossibility of obtaining information on the presence of chromosomal aberrations in the fetus at such an early gestational age, even those not detected by QF-PCR and those not yet detectable on ultrasound examination due to early gestational age. This also in consideration of the growing requests from patients who undergo coelocentesis for the diagnosis of monogenic diseases to also know the chromosomal structure of fetus. Analyses were conducted on coelomic fluid in a very early week of gestation in a pregnancy with previously detected bilateral thoracic effusion. Parents have given their written informed consent to publish their case.

## 2. Materials and Methods

The patient, 8 + 2 weeks pregnant, underwent coelomic fluid sampling in order to perform an early prenatal diagnosis for beta thalassemia. She was placed in the lithotomy position and the vagina and external genitalia were carefully cleansed with an antiseptic solution. A 5-MHz transvaginal ultrasound transducer, covered with sterile rubber, was then used for measurement of fetal crown–rump length and fetal heart rate, identification of the amniotic membrane, celomic space and yolk sac and diagnosis of any uterine anomalies. A 20-G needle was introduced transvaginally into the coelomic cavity, through a guide attached to the transducer and three consecutive samples of 0.2, 0.2 and 0.6 mL were aspirated into three different syringes (Supplementary Figure S1 and Supplementary Video S4). To minimize the risk of contamination, the third sample was used for diagnostic testing. Previous studies [13,14] reported the use of the 29-gauge atraumatic needle to reduce the complication rate of the punction in amniocentesis. CF is often very dense, due to the amount of macromolecules and mucus inside; therefore, with a 29-gauge needle nothing would be aspirated, due to density. Furthermore, with such a thin needle it would be difficult to direct it into the space, often millimetric, to be able to aspirate. The fetal heart rate was measured again immediately after the procedure. No local or general anesthesia was used. In all cases, ampicillin (1 g) was administrated intramuscularly, approximately 1 h prior to coelocentesis [3].

### 2.1. Molecular Analysis for Prenatal Diagnosis of Beta Thalassemia on CF

Prenatal diagnosis for beta thalassemia was carried out following the laboratory workflow for prenatal diagnosis of monogenic diseases on CF [12]. Paternal mutation was HBB:c.92 + 6T > C in heterozygosity and maternal mutation was HBB:c.93-21G > A in heterozygosity. Eleven fetal cells were selected by micromanipulation of coelomic fluid with LEICA AM 6000 micromanipulator (Leica Microsystems, Wetzlar, Germany), connected to an optical phase contrast microscope (40× magnification) and stored in PBS for a total volume of 4 µL. DNA was extracted by alkaline lysis of selected fetal cells [15,16,17]. Two PCR steps for DNA amplification were used. The first step was a multiplex PCR (30 cycles) using specific primers to identify mutations of beta globin gene (HBB: NM_000518.5) and no fluorescent primers to analyze Short Tandem Repeat (STR) markers for maternal contamination check. Two nested PCRs were performed, one for DNA target containing molecular defects causative of hemoglobinopathies using internal primers and the other one for maternal contamination analysis using fluorescent primers. In both reactions, 3 µL of first PCR product were used and 25 PCR cycles were performed. Sequencing analysis was performed in order to search for parental pathogenetic variants. In accordance with the guidelines for prenatal diagnosis, the research of parental mutations was carried on also with another molecular technique, SnapShot Multiplex analysis, which uses a single-tube reaction to detect single nucleotide polymorphisms at known locations. The chemistry is based on the dideoxy single-base extension of an unlabeled oligonucleotide primer; each primer binds to a complementary template in the presence of fluorescently labeled ddNTPs and DNA polymerase. The polymerase extends the primer by one nucleotide, adding a single ddNTP to its 3’ end. The fluorescence color readout reports which base was added.

Evaluation of maternal contamination in CF was performed by a very sensitive multiplex fluorescent PCR (QF-PCR), using 14 STR markers selected on the basis of their high heterozygosity index and located on chromosomes 13, 18, 21, X and Y [12].

### 2.2. Molecular Analysis for Prenatal Diagnosis of Chromosomal Aberrations on CF

We have used array-CGH performed on WGA from a few fetal erythroblasts in order to develop a new protocol for the early detection of chromosomal aberrations, also those not detected by QF-PCR and those not yet detectable on ultrasound examination due to early gestational age.

#### 2.2.1. Whole Genome Amplification

A group of two embryo-fetal erythroid precursor cells selected through a new micromanipulation procedure was processed using GenetiSure Pre-Screen kit (Agilent Technologies, Inc. 5301 Stevens Creek Blvd. Santa Clara, CA, USA) that includes QIAGEN REPLI-g Multiple Displacement Amplification (Qiagen—Hilden, Germany) to perform whole genome amplification. WGA is a method for robust amplification of entire genome, starting with nanogram quantities of DNA and resulting in microgram quantities of amplified products. Several methods have been developed for high-fidelity whole genome amplification, including PCR-based methods such as Degenerate Oligonucleotide PCR (DOP-PCR) and Primer Extension Preamplification (PEP), but the most commonly used method is the Multiple Displacement Amplification (MDA), a non-PCR-based amplification that uses the strand-displacement activity of DNA polymerases such as phi29 or Bst. In our workflow, we proceeded with MDA, using random hexamer primers, high-fidelity Phi29 polymerase and isothermal conditions; these conditions provide highly uniform amplification across the entire genome, with minimal locus bias [18,19]. The entire workflow was performed in accordance with the protocol provided by Agilent Technologies company. Human reference genomic DNA Female and Male (provided by Agilent) was processed simultaneously to the cell samples to create, in the step of array-CGH, differently labeled Cy5-Cy3 pairs. The products of WGA were tested, loading 1.5 µL on 1% agarose gel. Qubit Fluorometer and Quant-it dsDNA Broad Range Assay Kit were used to measure the concentration of amplified DNA. Samples with a value of concentration higher than 300 ng/µL were evaluated suitable for the following step of labeling for array-CGH.

#### 2.2.2. Quantitative Fluorescent PCR (QF-PCR) of WGA Products

In order to evaluate maternal contamination, sequence coverage over the genome, artifact formation and events of allelic dropout, WGA products were diluted at a concentration of 40 ng/µL to perform a quantitative fluorescent PCR by Elucigene QST*R kit—a highly multiplexed single tube assay comprising a total of 16 markers for the detection of the 3 most common autosomal aneuploidies in chromosomes 13, 18 and 21—and QST*R-XY kit—a 12-plex single tube assay for determination of sex chromosomal aneuploidies, including Klinefelter and Turner syndromes—(Elucigene Diagnostics—Citylabs, Nelson Street, Manchester, United Kingdom) [20]. This 12-plex QF-PCR enables the identification of the Amelogenin marker, which amplifies non-polymorphic sequences on the X (104 bp) and Y (110 bp) chromosomes, and of the non-polymorphic Y-specific SRY marker, which allows also gender determination. The QF-PCR products were separated in an ABI PRISM 3130 xl Genetic Analyzer and fragment analysis was performed with Gene Mapper 4.0 software (Applied Biosystems by Life Technologies LTD, Warrington, UK).

#### 2.2.3. Array Comparative Genomic Hybridization

DNA copy number variations (CNVs) were detected using the GenetiSure Pre-Screen Kit, in accordance with the specific protocol provided by the Company [21,22]. Sample vs. sample was hybridized and analyzed against both male and female reference samples, using different label with cyanine-3 (Cy3) and cyanine-5 (Cy5) dyes and combining as Cy3-Cy5 pair. The procedure uses random primers and the exo(-) Klenow fragment to differentially label amplified DNA samples with fluorescent-labeled nucleotides. Labeled DNA was purified using the purification columns provided by the Company and then hybridized onto 8 × 60 K GenetiSure Pre-Screen microarrays, characterized by a uniform, genome-wide probe coverage, with increased density on chromosomes 13, 18, 20, 21, 22 and X; specifically, this platform contains ~55,000 unique biological probes with a median probe spacing of ~50 kb. After incubation at 67 °C at 20 rpm for 2 h and washing, the slide was scanned. Set parameters are shown in Table 1. In our laboratory’s environment, the ozone level exceeds 5 ppb, so an ozone-barrier slide cover was placed on top of the array before scanning. The microarray TIFF images were loaded into the Agilent CytoGenomics software (version 3.0) for feature extraction and analyzed with Single Cell Recommended Analysis Method, provided by Agilent for analysis of single/few cell samples. The settings of this Aberration Detection Method are shown in Table 2.

## 3. Results

### 3.1. Molecular Analysis for Prenatal Diagnosis of Beta Thalassemia on CF

**Figure 1:**
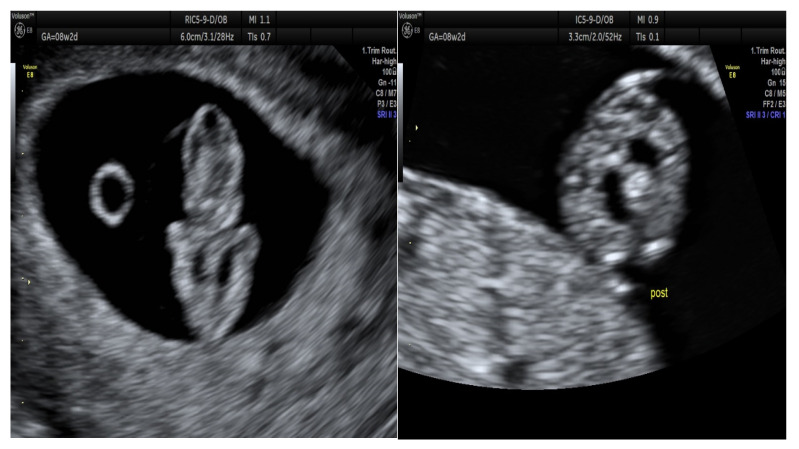
Transverse section of the chest of the embryo, bilateral thoracic effusion is displayed, the heart in the center. Pregnancy at 8 + 2 weeks of amenorrhea. Crown-rump length (CRL) 19.2 equal to 8 + 3 weeks. PRE-coelocentesis heart rate (HR) 181 bpm, POST-coelocentesis HR 176 bpm.

Coelocentesis was performed at 8 + 2 weeks of gestation and 950 μL of coelomic fluid was aspirated. Transvaginal ultrasound examination detected bilateral thoracic effusion; transverse section of the chest of the embryo with the abnormality is shown in Figure 1.

Embryo-fetal erythroid precursor cells were aspirated one by one using a 45-μm glass micropipette and placed into different drops of phosphate-buffered saline in the same Petri dish. Successively, the cells were aspirated and placed into 0.2-mL Eppendorf tubes and DNA was extracted by an alkaline procedure. The β-globin gene amplicons were sequenced with inner primers and run in ABI PRISM 3130 xl automated DNA sequencer. The QF-PCR products (2.5 μL) were mixed with 15 μL of formamide and 0.4 μL of Genescan-500 Liz containing the reference molecular size standard. Capillary electrophoresis for analysis of the fluorescent PCR products and size standards was performed with the ABI PRISM 3130 xl automated DNA sequencer and the GeneScan 4.0 software analyzer (Applied Biosystems).

**Figure 2:**
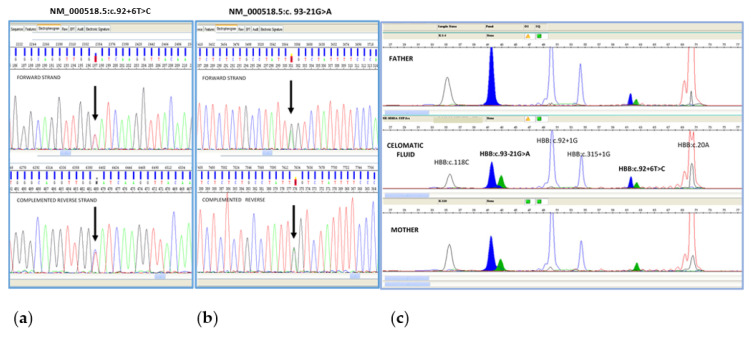
Fetal beta globin gene analysis: sequencing and SnapShot analysis are displayed. Sequencing analysis shows both paternal pathogenetic variant (NM_000518.5:c.92 + 6T > C) (**a**) and maternal pathogenetic variant (NM_000518.5:c.93-21G > A]) (**b**) SnapShot analysis shows the pathogenetic variant HBB:c.[92 + 6T > C] in heterozygous state in father, the pathogenetic variant HBB:c.[93-21G > A] in heterozygous state in mother and compound heterozygosity for paternal and maternal pathogenetic variants in fetus (**c**).

DNA analysis through QF-PCR showed no maternal DNA contamination, while both β-globin gene parental mutations were identified through sequencing and SnapShot analysis (Figure 2).

**Figure 3:**
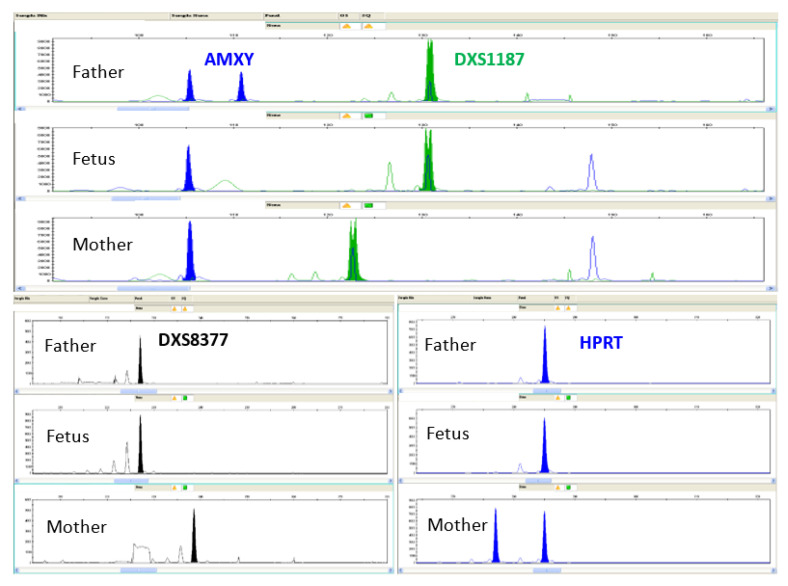
QF-PCR analysis, carried out on DNA extracted by alkaline lysis of eleven selected fetal cells, shows no informativeness on the arrangement of the X chromosomes. A monoallelic set-up of displayed Short Tandem Repeats (STRs) mapped on the X chromosome was found: AMXY, DXS1187, DXS8377, HPRT.

Furthermore, QF-PCR analysis was not informative on the arrangement of the X chromosomes (Figure 3); therefore, Array Comparative Genomic Hybridization was performed in order to obtain the molecular karyotype.

### 3.2. Molecular Analysis for Prenatal Diagnosis of Chromosomal Aberrations on CF

The new experimental application was based on a step of WGA starting from a few fetal erythroblasts collected from coelomic fluid, a step of evaluation of the sample’s suitability and a step of array-CGH performed on WGA products.

#### 3.2.1. Whole Genome Amplification

Two embryo-fetal erythroid precursor cells, selected through a second micromanipulation procedure, were processed by QIAGEN REPLI-g Multiple Displacement Amplification to perform whole genome amplification; 1.5 µL of product of amplification was loaded on 1% agarose gel to test the efficiency of WGA. The average concentration of amplified DNA, measured through Qubit Fluorometer and Quant-it dsDNA Broad Range Assay Kit, in according with the protocol provided by the Agilent Technologies, was 80.267 ng/µL, suitable for the following step of sample labeling for array-CGH.

#### 3.2.2. Quantitative Fluorescent PCR (QF-PCR) of WGA Products

**Figure 4:**
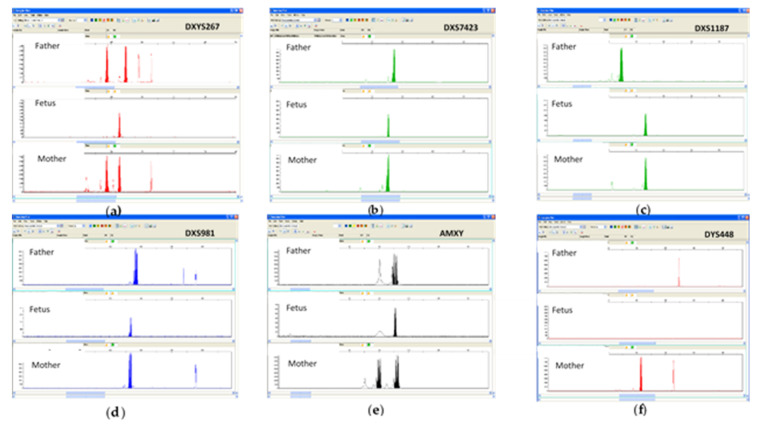
QF-PCR analysis performed on products of WGA carried out on DNA extracted by alkaline lysis of two selected fetal cells. A monoallelic set-up of all Short Tandem Repeats (STRs) mapped on the X chromosome was found. (**a**) DXYS267; (**b**): DXS7423; (**c**) DXS1187; (**d**) DXS981; (**e**) AMXY; (**f**) DYS44.

QF-PCR by Elucigene QST*R and QST*R-XY kit, performed on WGA products, is a supplementary step not included in the specific protocol provided by the Company, but in our experience, it adds more information to the procedure and shows the quality of WGA products. Evaluation by QF-PCR of WGA products excluded maternal contamination and showed a monoallelic pattern of all Short Tandem Repeats (STRs) mapped on the X chromosome, thus confirming our suspicion of a chromosomal aberration (Figure 4). The resulting coverage was considered sufficient for the next step, in accordance with the specific protocol provided by the Company, so array-CGH analysis was carried out.

#### 3.2.3. Array Comparative Genomic Hybridization

The Quality Control Report of array-CGH analysis conducted versus female (Supplementary Figure S1) and versus male (Supplementary Figure S2) are reported; all quality control parameters were good or excellent.

**Figure 5:**
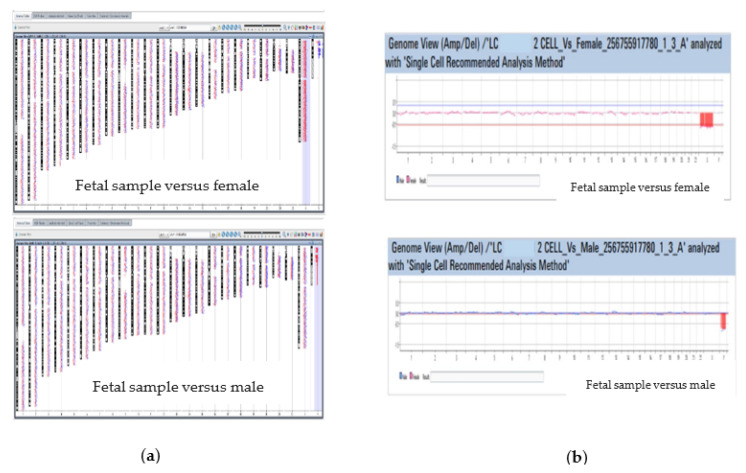
MultipleSample Triage View (**a**) and Single Cell Triage (**b**) are displayed.

The Single Cell Recommended Analysis Method, provided by the Company for analysis of single/few cell samples, was used. Multiple Sample Triage View and Single Cell Triage are shown in Figure 5.

Analysis conducted versus female showed the loss of one X chromosome, while analysis conducted versus male showed the loss of Y chromosome. These results are compatible with a monosomy X. The couple, after genetic counseling, decided to terminate the pregnancy in our hospital at 9 + 5 weeks by voluntary termination of pregnancy (VTP). Analysis of placental tissue after abortion confirmed the results obtained on coelomic fluid, both for thalassemia and monosomy X.

## 4. Discussion

In consideration of the precocity of coelocentesis, carried out at least 4 weeks earlier than CVS, the number of women who request this procedure is increasing, because of the precocity of the test. At our department, coelocentesis is used routinely as a diagnostic procedure for monogenic diseases in alternative to CVS or amniocentesis, if it is specifically requested by the woman.

Sampling and analysis of coelomic fluid between VII and IX week of gestation has proven to be a reliable and safe procedure of prenatal diagnosis in the case of monogenic diseases and for early detection of aneuploidies on chromosomes 13, 18, 21, X and Y by QF-PCR, but so far it is impossible to obtain a conventional karyotype because of the exiguity of fetal cells. We are trying to overcome this limit, also in consideration of the growing requests from patients who undergo coelocentesis for the diagnosis of monogenic diseases to also know the chromosomal structure of the fetus. The incidental detection of a monosomy X by QF-PCR in the reported early prenatal diagnosis for beta thalassemia on coelomic fluid represented the starting point for developing a new protocol for the early detection of chromosomal aberrations, even those not detected by QF-PCR and those not yet detectable on ultrasound examination due to early gestational age. Array-CGH analysis is a well-known method for definition of molecular karyotype; the application of a specific platform to WGA products starting from a few fetal erythroblasts collected from coelomic fluid is a new experimental application to overcome the impossibility of detecting chromosomal aberrations in the fetus at such an early gestational age. Therefore, the procedure reported in our manuscript may be considered as a method to be implemented for the early detection of chromosomal aberrations by the use of CGH-array with a specific platform.

This report underlines the importance of an early prenatal diagnosis and the countless potentials of array-CGH that could make the definition of molecular karyotype possible from few fetal cells, unlike conventional cytogenetic techniques that require a higher cellular content, available only at a later stage of gestation (XI-XIII weeks).

Ultrasound examination had revealed bilateral thoracic effusion, but this ultrasound sign could have gone unnoticed given the early gestational age. Molecular analyses performed on DNA extracted from coelomic fluid showed compound heterozygosity for paternal and maternal pathogenetic variants in β-globin genes, so the fetus was affected by beta thalassemia. Furthermore, QF-PCR analysis, performed in order to exclude maternal contamination in fetal sample, was not informative about the arrangement of the X chromosomes; therefore, molecular karyotype was obtained by Array Comparative Genomic Hybridization. This is the first report of a molecular karyotype obtained from two cells selected by micromanipulation of coelomic fluid and the first report of a molecular karyotype defined at such an early gestational age.

The use of an early chromosomal microarray analysis based on comparative genomic hybridization on coelomic fluid could be a valid technique that could allow the analysis of the whole chromosomal structure at the same time as the execution of molecular testing for monogenic diseases, carried out at an early gestational age. The application of this technology on coelomic fluid in the field of prenatal diagnosis has shown that this methodology could find useful application in the near future in the diagnosis of chromosomal abnormalities (as well as monogenic diseases), even if some problems still have to be solved) as the use of an automatic procedure for the isolation of fetal cells. Before the procedure could be transferred to routine diagnostics, a thorough validation must be performed on a considerable number of samples. Moreover, collected data must be validated on DNA extracted from well-known tissues and processed on a standard platform. The limitation of the procedure is mainly due to the phase of recognition and selection of fetal cells, which require a significant experimentation time; however, the data and results reported in this article may support the approach to this procedure. In addition, the possibility of performing the molecular karyotype on coelomic fluid could overcome the problem of the impossibility, at the current state of knowledge, of putting in culture the fetal cells present in the coelomic fluid to perform the conventional karyotype.

In conclusion, this procedure of sampling is attractive to couples at risk because it already provides prenatal diagnosis of monogenic diseases and probably soon chromosomal aberrations at least 4 weeks earlier than others traditional invasive procedures, reducing the anxiety of parents and providing the option for termination of pregnancy in case of affected fetuses at 8–10 week of gestation, which is less traumatic and safer than second-trimester surgical termination. However, it is necessary to increase the number of molecular karyotypes carried out on coelomic fluids and at the same time confirm the results on chorionic villi, amniotic fluid or peripheral blood after birth in order to validate the new procedure on a well-known and experimented workflow.

## Figures and Tables

**Table 1:** Settings of Scanner C scan.

Scanner C Scan Settings	
Dye channel	R + G (red + green)
Scan region	Agilent HD (61 × 21.6 mm)
Scan resolution	3µm
Tiff file dynamic range	16 bit
Red PMT gain	100%
Green PMT gain	100%
XDR	<noXDR>

**Table 2:** Single Cell Recommended Analysis Method.

Aberration Filter Settings for Single Cell Recommended Analysis Method
Minimum Size: 5 Mb
Minimum log_2_ ratio: 0.35 for amplification/gain −0.45 for deletion/loss

## Data Availability

All datasets on which the conclusions of the paper rely are available to editors, reviewers and readers without restriction and attached as figures and tables.

## Supplementary Materials

The following supporting information can be downloaded at: https://www.mdpi.com/article/10.3390/life13010020/s1. Figure S1: Ultrasound image of 8-week pregnancy undergoing coelocentesis, showing needle in coelomic cavity. Figure S2: FE QC Report vs. female is displayed. Figure S3: FE QC Report vs. male is displayed. Video S4: procedure of sampling of coelomic fluid.
