# Supplementary material for: Incidental Detection of a Chromosomal Aberration by Array-CGH in an Early Prenatal Diagnosis for Monogenic Disease on Coelomic Fluid
Source: Life (Basel). 2022 Dec 21;13(1):20. doi: 10.3390/life13010020 (PMC9863495; doi:10.3390/life13010020)
Supplement: Supplementary file 1 [file life-13-00020-s001.zip › Supplementary figure S1.pdf]

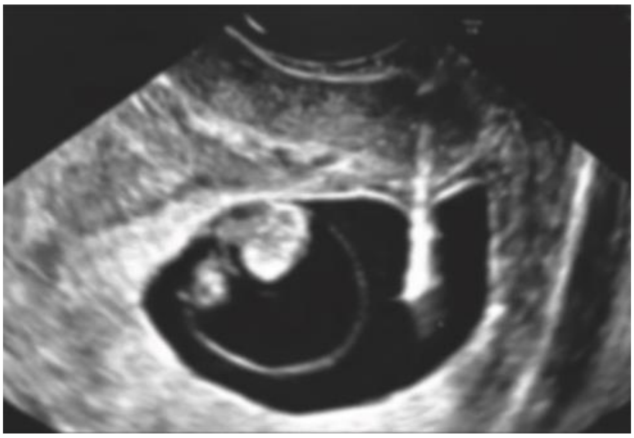

Supplementary Figure S1: Ultrasound image of 8-week pregnancy undergoing coelocentesis, showing needle in coelomic cavity.
