# Supplementary material for: Incidental Detection of a Chromosomal Aberration by Array-CGH in an Early Prenatal Diagnosis for Monogenic Disease on Coelomic Fluid
Source: Life (Basel). 2022 Dec 21;13(1):20. doi: 10.3390/life13010020 (PMC9863495; doi:10.3390/life13010020)
Supplement: Supplementary file 1 [file life-13-00020-s001.zip › Supplementary figure S2.pdf]

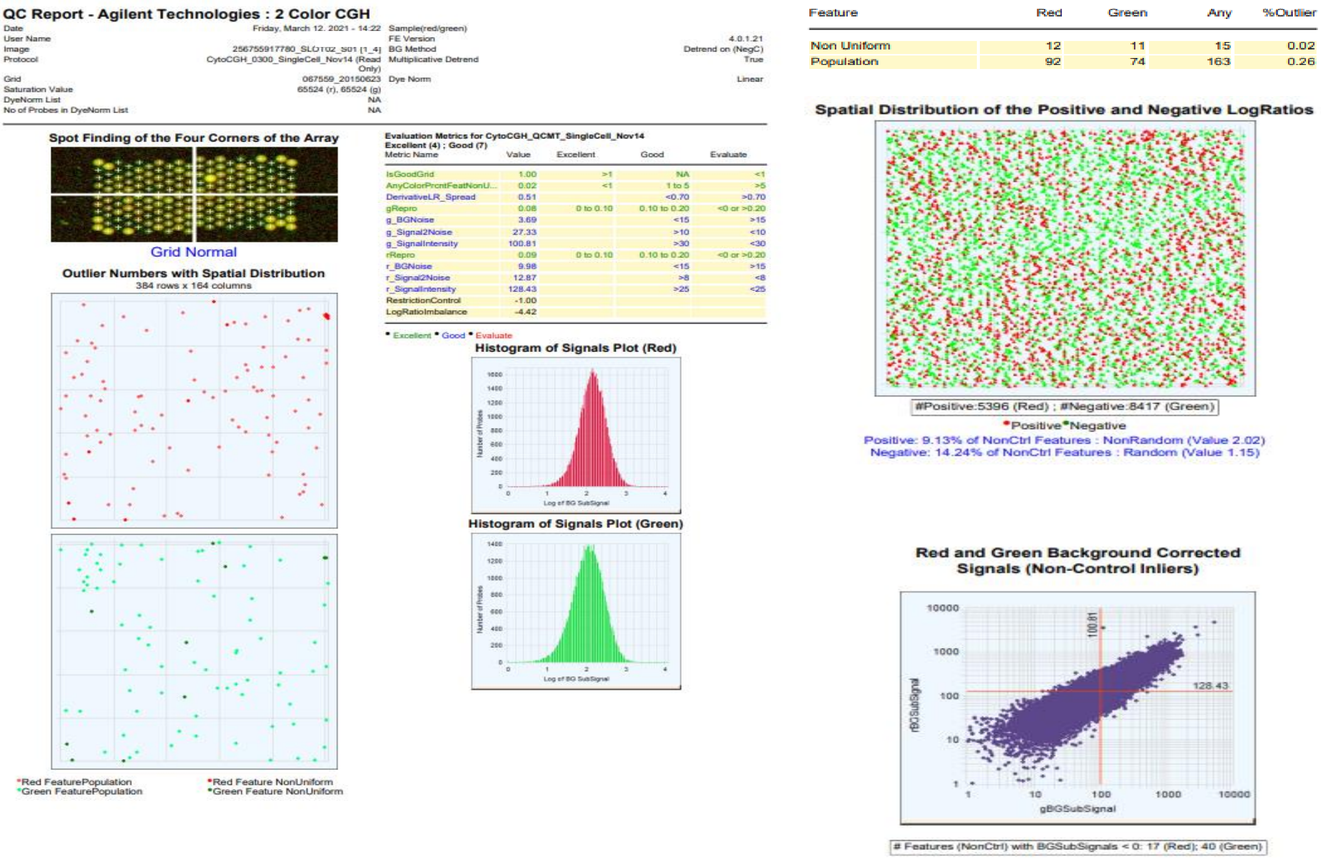

QC Report - Agilent Technologies : 2 Color CGH

Date	Friday, March 12, 2021 - 14:22	Sample(red/green)	
User Name		FE Version	4.0.1.21
Image	256755917780_SLOT02_S01 [1_4]	BG Method	Detrend on (NegC)
Protocol	CytoCGH_0300_SingleCell_Nov14 (Read Only)	Multiplicative Detrend	True
Grid	067559_20150623	Dye Norm	Linear
Saturation Value	65524 (r), 65524 (g)		
DyeNorm List	NA		
No of Probes in DyeNorm List	NA		

Evaluation Metrics for CytoCGH_QCMT_SingleCell_Nov14
Excellent (4) ; Good (7)

Metric Name	Value	Excellent	Good	Evaluate
IsGoodGrid	1.00	>1	NA	<1
AnyColorPrcntFeatNonU...	0.02	<1	1 to 5	>5
DerivativeLR_Spread	0.51		<0.70	>0.70
gRepro	0.08	0 to 0.10	0.10 to 0.20	<0 or >0.20
g_BGNoise	3.69		<15	>15
g_Signal2Noise	27.33		>10	<10
g_SignalIntensity	100.81		>30	<30
rRepro	0.09	0 to 0.10	0.10 to 0.20	<0 or >0.20
r_BGNoise	9.98		<15	>15
r_Signal2Noise	12.87		>8	<8
r_SignalIntensity	128.43		>25	<25
RestrictionControl	-1.00			
LogRatioImbalance	-4.42			

Feature	Red	Green	Any	%Outlier
Non Uniform	12	11	15	0.02
Population	92	74	163	0.26

Supplementary Figure S2. FE QC Report vs female is displayed.
